# Current Prevalence Pattern of Hypertension in Nigeria: A Systematic Review

**DOI:** 10.1371/journal.pone.0140021

**Published:** 2015-10-13

**Authors:** James Tosin Akinlua, Richard Meakin, Aminu Mahmoud Umar, Nick Freemantle

**Affiliations:** 1 Department of Primary Care and Population Health, University College London Medical School (Royal Free Campus), Rowland Hill street, London, NW3 2PF, United Kingdom; 2 College of Science and Technology, University of Salford, Salford Crescent, Manchester, M5 4WT, United Kingdom; University of Perugia, ITALY

## Abstract

**Background:**

The global burden of hypertension and other non-communicable diseases (NCDs) is rapidly increasing, and the African continent seems to be the most affected region in the world. The prevalence of hypertension in Nigeria forms a substantial portion of the total burden in Africa because of the large population of the country currently estimated to be over 170 million.

**Objective:**

The purpose of this systematic review is to summarise up to date data on the prevalence and distribution of hypertension in Nigeria from prevalence studies.

**Methods:**

A search of the following databases: PubMed, EMBase and WHO cardiovascular InfoBase from 1968 till date was conducted to identify studies which provide estimates of prevalence of hypertension in Nigeria.

**Results:**

The search yielded a total of 1748 hits from which 45 relevant studies met the inclusion criteria for the review. The overall crude prevalence of hypertension ranged from 0.1% (95%CI:-0.1 to 0.3) to 17.5% (95% CI: 13.6 to 21.4) in children and 2.1% (95%CI: 1.4 to 2.8) to 47.2% (95%CI: 43.6 to 50.8) in adults depending on the benchmark used for diagnosis of hypertension, the setting in which the study was conducted, sex and ethnic group. The crude prevalence of hypertension ranged from 6.2% (95%CI: 4.0 to 8.4) to 48.9% (95%CI: 42.3 to 55.5) for men and 10% (95%CI: 8.1 to 12) to 47.3% (95%CI: 43 to 51.6%) for women. In most studies, prevalence of hypertension was higher in males than females. In addition, prevalence across urban and rural ranged from 9.5% (95%CI: 13.6 to 21.4) to 51.6% (95%CI: 49.8 to 53.4) and 4.8% (95%CI: 2.9 to 6.7) to 43% (95%CI: 42.1 to 43.9) respectively.

**Conclusions:**

The prevalence of hypertension is high among the Nigerian population. Appropriate interventions need to be developed and implemented to reduce the preventable burden of hypertension especially at Primary Health Care Centres which is the first point of call for over 55% of the Nigerian population.

## Introduction

The “big three” infectious diseases- Malaria, tuberculosis and HIV/AIDS coupled with childhood and maternal mortality are still the prominent causes of mortality within Nigeria and Africa as a whole [1, 2]. However, globally, the burden of hypertension and other non-communicable diseases (NCDs) is rapidly increasing, and the African continent may be the most affected region in the world [3]. The United Nations (alongside other major public health stakeholders) has declared NCDs a cause for global concern [3, 4].

It is estimated that hypertension affects about 1 billion people all over the world and it is the main risk factor for many other cardiovascular diseases [4, 5, 6, 7]. The prevalence of hypertension in Nigeria may form a substantial proportion of the total burden in Africa because of the large population of the country currently estimated to be over 170 million [4, 7,8].

With an increasing adult population and changing lifestyle of Nigerians, the burden of hypertension may continue to increase as time unfolds [9, 10]. In suggesting an evidence-based context for government and other health policy planners on strategies to reduce this burden in low-resource settings like Nigeria, it is important to have detailed up to date information on the prevalence of hypertension in order to match this with available resources.

In Nigeria, the last two decades has seen a rise in the number of prevalence studies concerning hypertension and other non-communicable diseases [11–16]. Similarly, a number of systematic reviews of the various prevalence studies on hypertension have been done, the most recent being the study by Adeloye et al [4, 11,17]. The study by Adeloye et al [4] was quite detailed in its presentation and analysis in that, it conducted several appropriate meta-analysis of data points from different studies as well as epidemiological models to predict prevalence of hypertension in the future.

However, the study did not consider studies conducted in different Nigerian health care settings i.e. primary, secondary and tertiary health centres. In addition, studies on prevalence of hypertension in children were not included. Given the potential importance of different health care settings in the prevention, treatment and control of hypertension among all age groups, it is pertinent to have a broader picture of the prevalence of hypertension in Nigeria. Therefore, this study builds up on the robust work of Adeloye et al to include prevalence studies conducted in hospitals and among children to inform further research and actions.

## Country Profile

Amongst black nations of the world, Nigeria is the most populous and it is the most populated country in Africa with a population of over 160 million people [16, 18]. About 48% of this large population reside in cities while the remaining 52% reside in rural areas. Nigeria has over 250 ethnic groups [18, 19].

**Fig 1:**
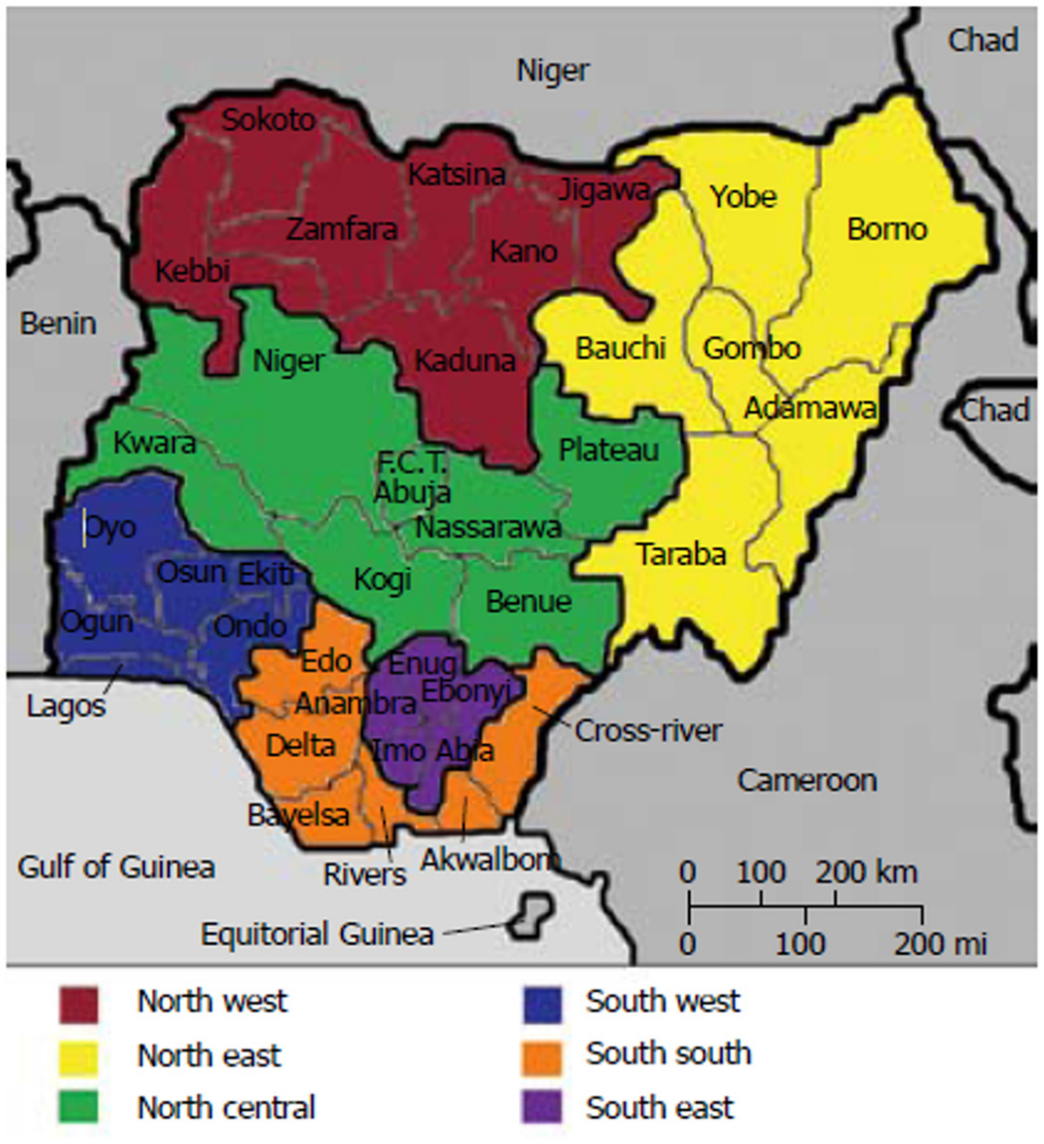
Map of Nigeria showing the 6 geo-political zones, 36 states and federal capita territory (adapted from [11].

There is a democratically elected government and parliament at the three tiers of government namely: federal, state and local government within thirty six states, 6 geo-political zones and a federal capital territory [19] (see Fig 1).

“According to UNDP (2008), the literacy rate in Nigeria is 69.1%, with poverty rate of under $1.25 per day for 64.4% of the population and under $2 for 83.9%” (pg 25) [19]. Life expectancy at birth was 53 years old for males and 55 years for females in 2012. The Total expenditure on health per capita (Intl $, 2012) was 161. Total expenditure on health as % of GDP (2012) was 6.1 [20].

## Methods

### Eligibility Criteria

Eligibility criteria and the protocol adopted for the study was patterned after that recommended by the National health and medical research council document 1999 for systematic reviews of cross-sectional studies [21].

### Types of studies

Cross Sectional studies conducted in rural or urban areas (including hospital based studies) with study sample size of 400 or more were selected for this review. It was calculated that to be able to estimate a prevalence of between 10–50% with a 5% margin of error and 95% level of confidence, a minimum sample size of 400 was required [22]. Studies included must have employed random sampling of a well-defined population or studies using whole populations [23]. The Response rate should be greater than 70% and the study should be conducted on Nigerians only [24]. In addition, studies included should have reported prevalence of hypertension (age adjusted or unadjusted) [25].

### Type of participants/case definitions

Studies of prevalence of hypertension in all age groups and both sexes with standard methods of measuring blood pressure were considered [26]. For studies conducted on younger children (Pre-school age group); studies with hypertension defined as “systolic or diastolic blood Pressure greater than or equal to the 95th percentile for age and gender measured on at least three separate occasions” were included (pp 1) [27].

For studies conducted on adolescents and adults, studies with hypertension defined as at least 140 mmHg for SBP and 90 mmHg for DBP were included. This diagnostic criteria/Case definition is based on the definitions of hypertension by the; Joint National Committee on prevention, detection, evaluation, and treatment of high blood pressure (JNC6 and JNC7); the 1999 WHO/International Society of Hypertension (WHO/ISH) definitions and classification of blood pressure levels [28,29,30] and the 2003 WHO/ISH Statement on Management of Hypertension [31]. These organisations all put their threshold for hypertension at 140/90mmHg.

However, it is worthy of note that many prevalence studies conducted before 1999 used a cut-off point of ≥160/95mmHg for diagnosing high blood pressure. Because the aim of this study is not to synthesize the data but to appraise the available data from different studies to give a broad picture of the problem, all these studies were included as well, and comparison made between studies that used different definitions.

## Sources of Information

All Studies were found by searching through the electronic databases (MEDLINE AND EMBASE), WHO cardiovascular InfoBase and looking through reference list of articles identified for relevant articles not indexed in the databases. Government bulletins and documents such as the federal Ministry of health of Nigeria’s National Non Communicable Disease surveys were also assessed. No language limits were applied to the search strategy. The electronic search was applied to MEDLINE (1966 till present date) and adapted to EMBASE (1980 till present date). The current content of the WHO cardiovascular InfoBase was also reviewed. The Last search was conducted on the 17^th^ of February 2015.

## Search Strategy

The following search terms were used to search all databases: prevalence, estimate*, hypertension, “blood pressure”, “raised blood pressure”, “high blood pressure”, Nigeria*. For the purpose of comprehensiveness of search, both free text terms and medical subject headings search was done in both databases. In addition, both free text terms and medical subject headings were exploded and truncated to capture as many articles as possible. No language, publication year or restriction of publication status was imposed in the search strategy. The limit “non-human” was included in the search strategy to exclude all articles conducted in non-humans. See S1 appendix for details of search strategy for EMBASE (ovidSP) and MEDLINE (ovidSP).

## Study Selection

**Fig 2:**
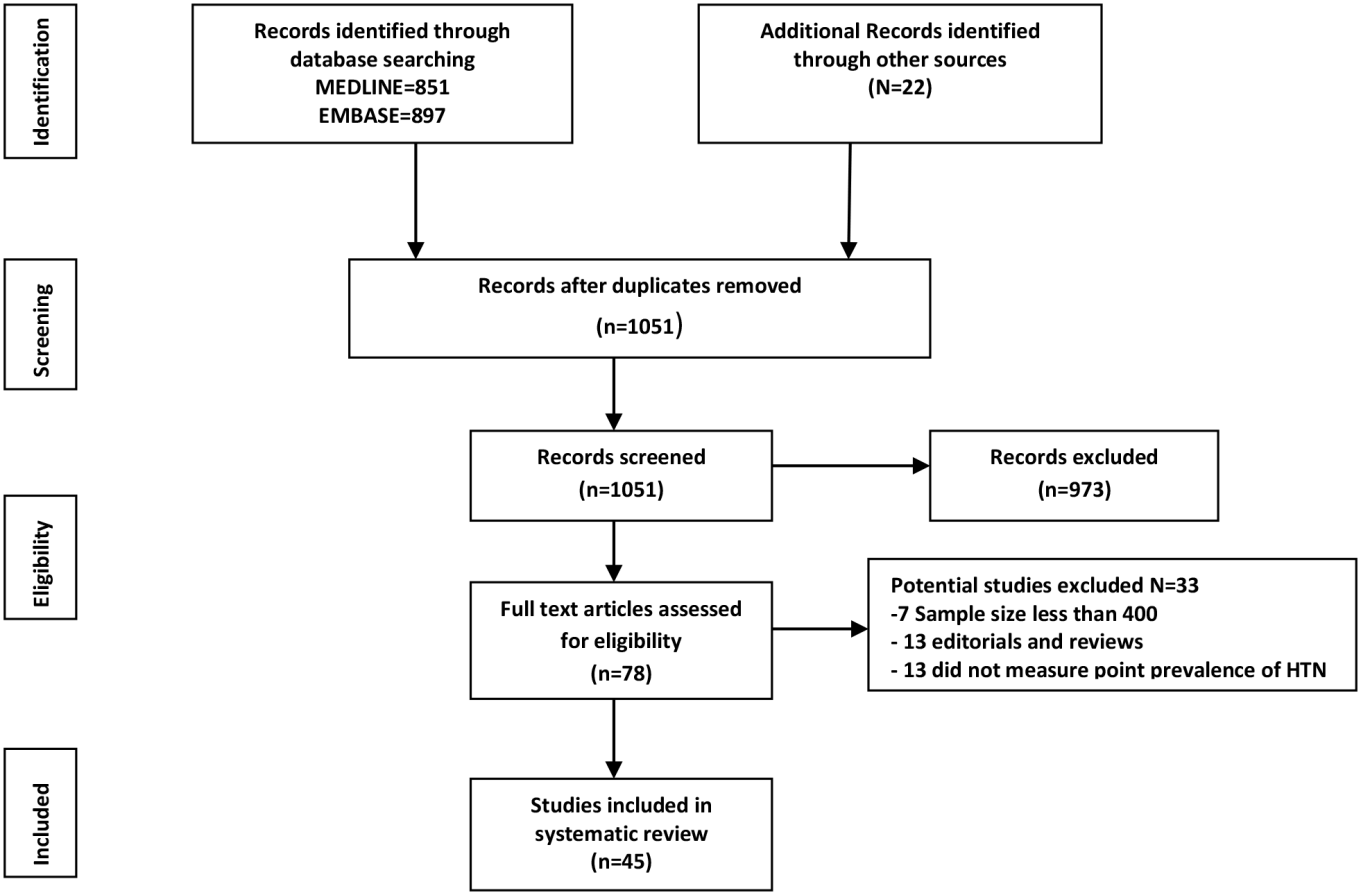
Study selection and extraction adapted from [32].

The process of study selection and extraction is presented in a PRISMA flow chart [32], in Fig 2 below. The search returned 1748 publications from MEDLINE (851), EMBASE (897), two (2) government documents from the federal ministry of health, Nigeria, ten (10) from WHO cardiovascular InfoBase and ten (10) relevant articles from references of already identified articles. After removing duplicates and applying the limit “humans only”, one thousand and fifty-one (1051) studies remained. After screening titles and abstracts for relevance i.e. prevalence studies conducted primarily on Nigerians, 973 studies were excluded. Therefore 78 full texts were assessed and after applying the eligibility criteria and quality criteria, a further 33 studies were excluded. Finally, a total of 45 studies [33–78] were included in the review. A table containing list of all potentially-eligible studies with reasons for exclusion is presented in S2 Appendix.

## Data Collection Process

Data from included studies were extracted in duplicate by two authors (JTA and AMU). Any uncertainties were resolved by discussion. The methods were developed and piloted on first 10 papers using a modified Data Extraction Template. Data extracted included the following items: year of survey, state and region where the study was conducted; age of participants; sampling methods; response rate; sample size, health-care setting, and BP definition used for hypertension. In addition, where available, prevalence of high blood pressure (adjusted and unadjusted) and percentage of people with high blood pressure who had been diagnosed before and on treatment were also retrieved.

## Risk of Bias in Individual Studies

Because the review question is that of frequency of a health problem, the risk of bias in studies focused on 3 main areas: Sample frame, Case ascertainment and adequate response rate [21]. These areas of bias were addressed in the eligibility criteria hence to ascertain the validity of eligible studies, the eligibility criteria described above which were based on the recommendations of the NATIONAL HEALTH and MEDICAL RESEARCH COUNCIL document were applied [21].

## Synthesis and Statistical Methods

The principal purpose of the study was to appraise the available data to give a broad understanding of the scale of the problem of hypertension and the variation between different settings and populations in Nigeria.

Because of the differences in design and context of different studies, and our interest in the differences between the estimates of hypertension prevalence in different contexts, we did not undertake quantitative synthesis of the results of the included studies (Meta analysis) but conducted a narrative synthesis.

In addition, we conducted limited numbers of new Chi Square tests to examine differences between studies addressing our objectives.

## Results

### Description of studies

Forty-four (44) independent studies [27, 33–77] were included in this review. These studies were published between the years 1968 and 2015. 41 studies [27, 33–77] were conducted across the 6 geopolitical zones of Nigeria and 3 studies [37, 73, 75] were conducted nationally. The numbers of those conducted in the zones are as follows: south- west 15[35–48,54–59,64,67,68]; south-east 8 [27,42,47,51–53,63,66,74]; south-south 10[33,45,60–62,65,70–72,76]; north-central 4 [34,46,48,51]; north-east 1[49] and north-west 4 [39,40,50,69].

Table 1 shows the characteristics of all studies included for the review including crude prevalence of hypertension reported for each study. The included studies were generally large; community based [33–52,54–63,68–70,73–76]; schools based [27,65–67]; hospital based [36,53,69]; and workers (civil-servants and factory workers)[64,71,72]. Sample sizes of studies ranged from 400 [33] to 13,591 [73]. The age of participants ranged from 0 to 110 years old. The setting where the studies were conducted included rural (14 studies), urban (22 studies including urban slums and semi-urban) and mixed (8 studies) i.e. urban and rural at the same time.

**Table 1:** Characteristics of all studies included in the systematic review.

							CRUDE PREVALENCE %		
FIRST AUTHOR	REGION	BP cut-off	TARGET POPULATION	SETTING	Age (years)	SAMPLE SIZE (Response Rate %)	OVERALL (95%CI)	MEN(95%CI)	WOMEN(95%CI)
Odetunde,2014^27^	SE	>95^th^ P^α^	school based	Urban	2–5	630(100)	1.9(0.83–2.96)	1.3(0.4–2.1)	0.63(0.01–1.3)
Abegunde,2013^35^	SW	140/90	community based	urban/rural	60–110	630(98.4)	36.5 (32.7–40.3)	NA	NA
Ekore,2009^36^	SW	140/90	hospital based	Urban	18–44	405(100)	30.6(26.1–35.1)	34.4(26.9–41.9)	28.3(22.7–33.9)
Cooper,1997^37^	national	140/90	community based	Rural	25–74	2509(96)	14.5(13.1–15.9)	14.7(12.7–16.7)	14.30(12.4–16.2)
Daniel,2013^38^	SW	140/90	community based	urban slum	20–81	964(100)	38.2(35.1–41.3)	44.5(39.1–49.9)	34.9(31.1–38.6)
Makusidi,2013^39^	NW	140/90	community based	Urban	15–80	535(99)	30.2(26.3–34.1)	NA	NA
Murthy,2013^73^	national	140/90	community based	urban/rural	≥40	13,591(99.4)	44.7(43.5–46.3)	42.6(40.9–44.4)	46.8(45.3–48.4)
Isezuo,2011^40^	NW	140/90	community based	Rural	15–65	782(100)	24.8(21.8–27.8)	24.9(20.7–29.1)	23.60(19.2–27.9)
Olatunbosun,2000^41^	SW	160/95	community based	Urban	≥ 18	998(100)	10.3(8.4–12.2)	13.9(11.1–16.7)	5.3(3.2–7.5)
okpechi,2013^42^	SE	140/90	community based	urban/rural	≥18	2983(99)	31.4(29.7–33.1)	34.9(32.1–37.4)	28.1(25.9–30.3)
Oladapo,2010^43^	SW	140/90	community based	Rural	18–64	2000(100)	20.8(19.0–22.6)	21.1(18.4–23.8)	20.5(18.1–22.9)
Ige,2013^44^	SW	140/90	community based	Urban	28–50	525(95.9)	21.5(17.9–25.0)	21.9(6.9–26.8)	21.1(16.1–26.1)
Ekanem,2013^45^	SS	140/90	community based	Urban	15–65	442(99)	47.0(42.3–51.7)	30.1(24.1–36.1)	16.8(11.8–21.8)
Hendriks,2012^46^	NC	140/90	community based	Rural	≥18	2678(99)	19.3^#^(17.3–21.3)	NA	NA
Ulasi,2010^47^	SE	140/90	community based	urban/rural	25–65	1458(75.8)	32.8(30.4–35.2)	NA	NA
Johnson,1971^48^	SW	160/95	community based	Urban	10–102	1392(100)	8.9(7.4–10.4)	7.9(5.8–10)	9.9(7.8–12.0)
Okesina,1999^49^	NE	140/90	community based	Rural	≥18	500(100)	15.2(12.0–18.3)	19.1(14.5–23.7)	10.3(6.3–14.3)
Ejike,2010^34^	NC	>95th P^α^	community based	urban/rural	13–18	843(100)	10.1(8.1–12.1)	9.0(6.4–11.7)	11.3(8.2–14.4)
Mijinyawa,2008^50^	NW	140/90	community based	Urban	13–19	1000(100)	7.2(5.6–8.9)	6.2(4.0–8.4)	7.7(5.3–10.1)
Ekezie,2011^51^	NC/SE	140/90	community based	urban/rural	20–80	567(82.7)	21.7(18.3–25.1)	NA	NA
Ulasi,2011^52^	SE	140/90	community based	Urban	≥20	731(94.1)	42.2(38.5–35.9)	46.3(43.6–48.9)	37.7(32.5–42.9)
Ike,2009^53^	SE	140/90	hospital based	Urban	≥20	1360(100)	18.4(17.5–19.2)	17.2(16.1–18.3)	18.9(17.5–20.3)
Adedoyin,2008^54^	SW	140/90	community based	semi urban	21–100	2097(92.3)	36.6(34.5–38.7)	36.8(33.6–39.9)	36.4(33.7–39.1)
Lawoyin,2002^55^	SW	160/95	community cohort	Urban	≥18	2144(99.4)	12.4(11.0–13.8)	12.1(10.6–14.0)	12.7(10.4–13.6)
Adebayo,2013^56^	SW	140/90	community based	Rural	15–90	1000(100)	26.4(23.7–29.1)	27.3(23.3–31.3)	25.4(21.6–29.2)
Ekpeyong,2012^76^	SS	140/90	community based	Rural	18–60	2780(96.3)	14.4(13.1–15.7)	12.6(10.9–14.3)	12.2(10.4–13.9)
Suleiman,2013^33^	SS	140/90	community based	Rural	≥20	400(100)	15(11.5–18.5)	18.8(12.7–24.9)	12.5(8.3–16.7)
Oluyombo,2014^57^	SW	140/90	community based	semi urban	≥18	750(89.8)	47.2(43.6–50.8)	48.9(42.3–55.5)	47.3(43.0–51.6)
Okpara,2015^48^	NC	140/90	Community based	Urban	≥16	471(100)	15.7(12.4–18.9)	16.5(12.4–20.6)	14.1(8.6–19.6)
Ezenwaka,1997^59^	SW	140/90	community based	urban/rural	≥55	500(100)	30(25.9–34.0)	25.8(19.8–31.8)	36.6(31.1–42.1)
Andy,2012^60^	SS	140/90	community based	Rural	≥18	3869(96.7)	23.6(23.3–24.9)	31.2(28.9–33.5)	18.1(15.8–20.4)
Onwuchekwa,2012^61^	SS	140/90	community based	Rural	≥18	1078(95)	18.3(15.9–20.6)	NA	NA
Omuemu,2007^62^	SS	140/90	community based	Rural	≥18	590(98)	20.2(16.9–23.4)	26.2(21.2–31.2)	13.2(9.3–17.1)
Onwubere,2011^63^	SE	140/90	community based	Rural	40–60	858(70.4)	46.4(43.1–49.7)	50.2(43.9–56.4)	44.8(40.9–48.7)
Ogunlesi,1991^64^	SW	160/95	male factoryworkers	Urban	≥18	541(100)	8(5.7–10.3)	NA	NA
Okpere,2013^65^	SS	>95th P^α^	school based	Urban	10–17	820(100)	3.2(1.9–4.4)	3.3(1.5–5.1)	3.1(1.4–4.8)
Ujunwa,2013^66^	SE	>95th P^α^	school based	Urban	10–18	2694(100)	5.4(4.5–6.3)	3.8(2.8–4.8)	6.9(5.6–8.2)
Oyewole,2012^67^	SW	>95th P^α^	school based	Urban	12–18	1638(100)	0.1(-0.1–0.3)	0.1(-0.2–0.33)	0.1(-0.2–0.33)
Akinkugbe,1968^68^	SW	140/90	community based	Rural	≥18	3602(100)	10.1(9.1–11.1)	9.1(8.1–10.1)	11.2(10.2–12.2)
Jain,1977^69^	NW	160/95	hospital based	Urban	≥18	2950(99.7)	3.8(3.1–4.5)	2.9(2.2–3.6)	4.9(4.2–5.6)
Oviasu,1977^70^	SS	160/100	community based	Rural	≥19	1482(96.8)	2.1(1.4–2.8)	2.8(2.1–3.5)	0.5(0.2–2.1)
Oviasu,1980^71^	SS	140/90	civil servants	Urban	≥18	1265(99)	13.3(11.4–15.2)	14(12.1–15.9)	10(8.1–12.0)
Idahosa,1985^72^	SS	140/90	civil servants	Urban	≥18	1450(98.2)	15.1(13.3–16.9)	NA	NA
Akinkugbe,1997^75^	National	160/95	community based	urban/rural	≥18	4930(98.4)	11.2(10.3–12.1)	NA	NA

^#^ = age standardised prevalence rate;

NA = not available;SW = south west;SE = south east;SS = south south;NC = north central;NW = north west;NE = north east;α = 95^th^ percentile

Only 5 studies [27,34,65–67] were conducted on children between 0 to18 years old. Although the search yielded more studies on this age group, most were concerned with pattern of blood pressure in this age group and not prevalence of hypertension.

### Diagnosis of hypertension

**Fig 3:**
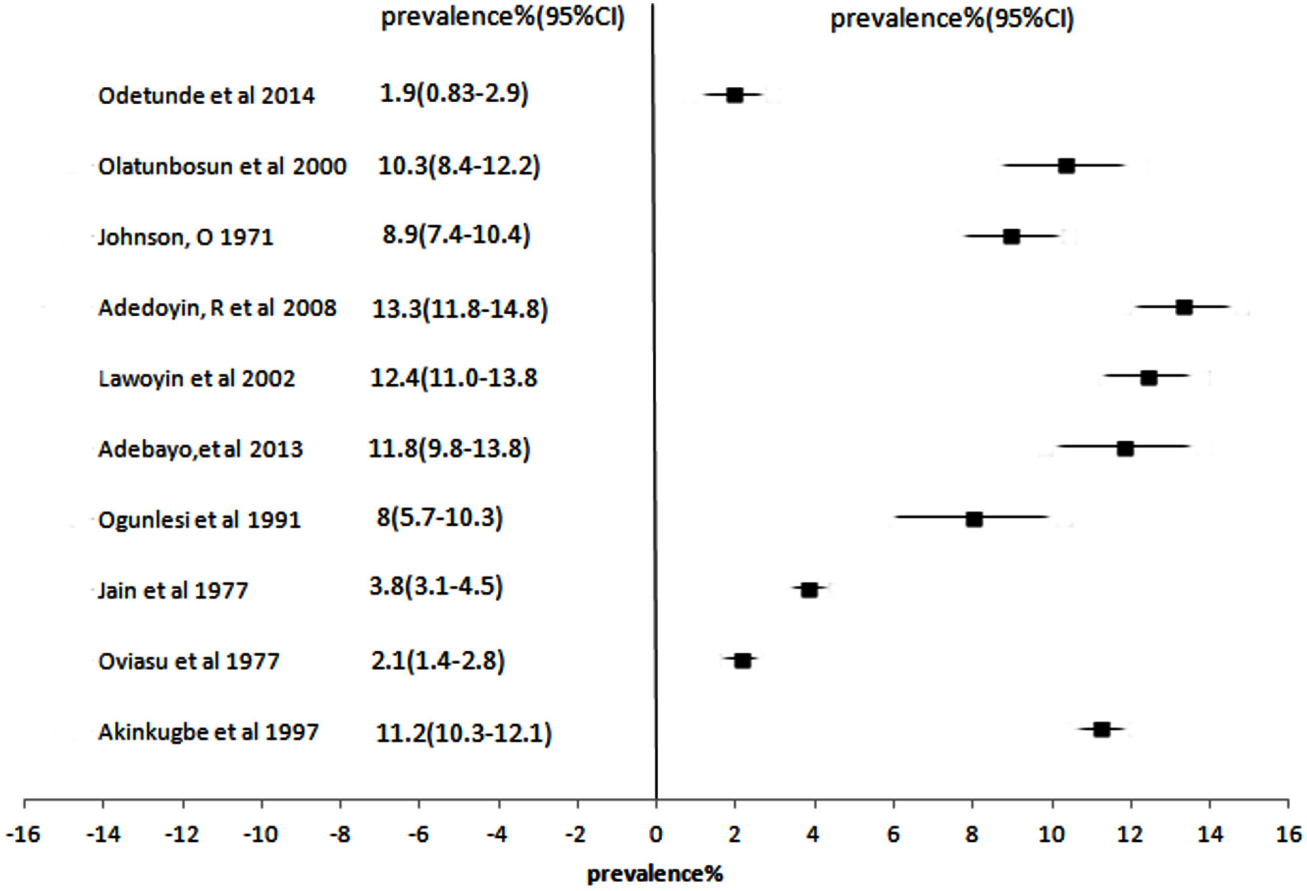
Forest Plot showing prevalence of hypertension% & 95%CI across studies that used BP cut-off ≥ 160/90mmHg.

Fig 3 illustrates the prevalence of hypertension for 8 studies [41,48,54–56,64,69,75] that used 160/95mmHg as their benchmark for diagnosing hypertension. Only one study used 160/100mmHg as cut-off [70]. Five studies [27,34,65–67] used the 95^th^ percentile value for age, sex and height as cut-off for hypertension in preschool and adolescents below 18 years of age. The remaining 32 studies used 140/90mmHg as cut-off.

### Prevalence of hypertension amongst children

Overall Crude Prevalence of hypertension recorded in studies conducted on children less than 18 years old [27,34,65–67] ranged from 0.1% (95%CI:-0.1 to 0.3) to 17.5% (95% CI: 13.6 to 21.4). The distribution of hypertension differed highly significantly across different studies conducted on children in Nigeria, χ^2^ (df, 5) = 155.686, *p* < 0.0001.

**With respect to settings (rural/urban):** The highest crude prevalence of hypertension 17.5% (95% CI: 13.6 to 21.4) was recorded in an urban area in the North Central zone. Similarly, the lowest prevalence 0.1% (95%CI:-0.1 to 0.3) was recorded in an urban area in the South West. However, the only study that compared adjusted prevalence between rural and urban children showed a slightly higher value for urban compared to rural (17.5% versus 4.6%).

**With respect to sex:** where reported the overall prevalence in males and females was very similar.

**With respect to increasing age:** The 2 studies done in the South East zone showed substantial differences in the crude prevalence (5.4% versus 1.9%). But the crude prevalence of 5.4% (95%CI: 4.5 to 6.3) was for the age group 10–18 years old while 1.9% (95%CI: 0.83 to2.96) was for age group 2–5 years. The prevalence recorded for 10–18 years old in the south-east zone study is relatively similar to the study conducted in Kano (North-West zone) which showed an overall prevalence of 7.2% (95%CI: 5.6 to 8.9) using BP cut off of 140/90mmhg amongst children aged 13–19 years old.

### Prevalence of hypertension amongst adults

The overall crude prevalence for studies conducted on adults aged 18 years and above ranged from 2.1% (95%CI: 1.4 to 2.8) to 47.2% (95%CI: 43.6 to 50.8).

**With regards to sex:** crude prevalence of hypertension ranged from 2.8% to 13.9% and 0.5% to 12.7% for males and females respectively in studies that used the BP benchmark of 160/95mmHg. In studies that used BP benchmark of 140/90mmHg crude prevalence rate of hypertension ranged from 6.2% to 48.9% and 10% to 47.3% for males and females respectively. Where male and female data are available irrespective of BP cut-off, overall crude prevalence rates were generally higher in males than in females (22 studies reported higher prevalence in males compared to females while 11 studies had higher prevalence in females compared to males. However, based on BP cut-off of 160/95mmHg, more studies had higher crude prevalence in females compared to males.

**Fig 4:**
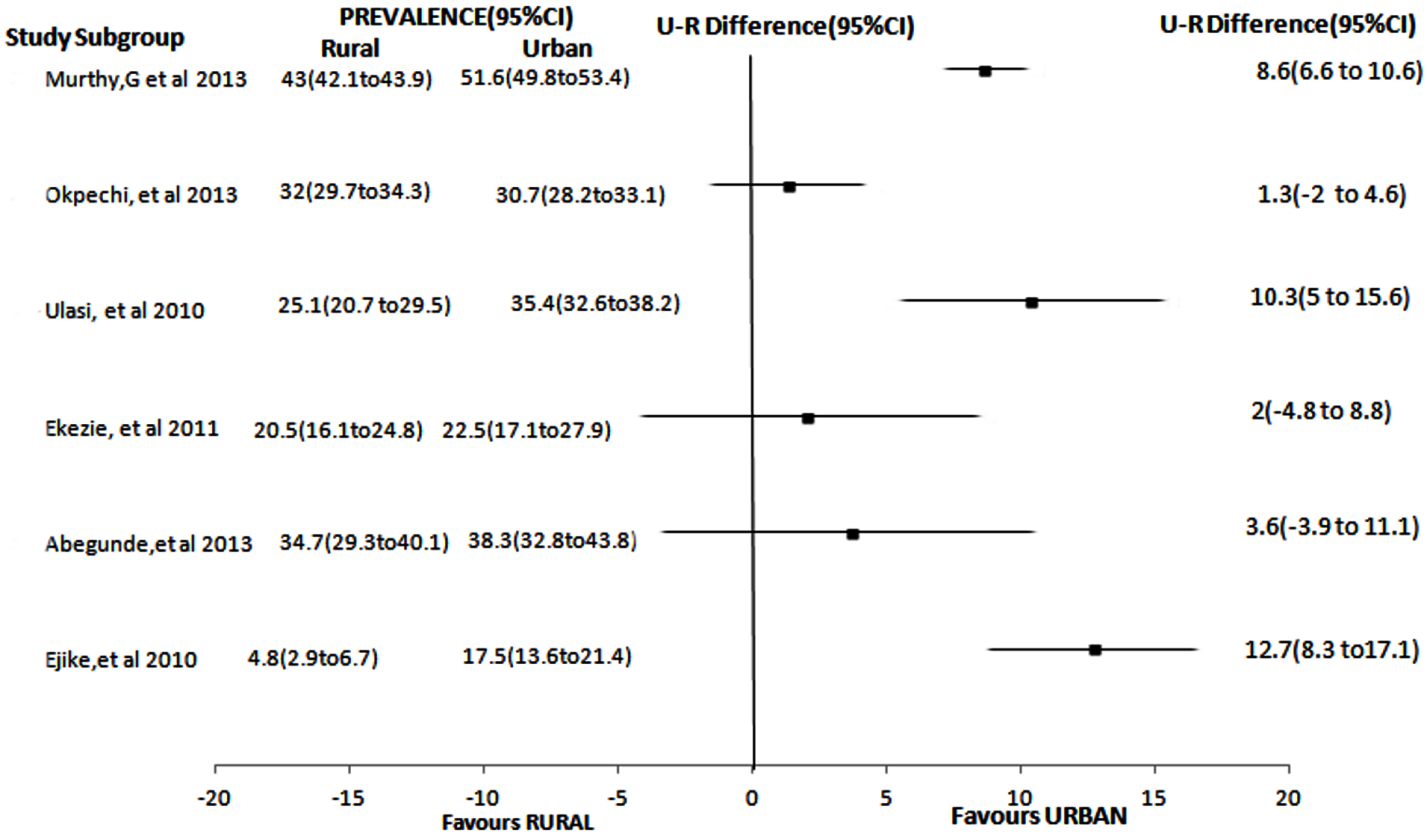
Forest Plot showing U-R difference of prevalence of hypertension in studies comparing rural and urban populations. U-R difference = Urban-Rural difference

**With regards to settings (Urban/Rural):** Fig 4 illustrates rural versus urban and urban-rural difference of crude prevalence in 6 available mixed studies (i.e. rural and urban in the same study) [34,35,42,51,52,73]. It is clearly demonstrated in the forest plot that using urban-rural differences in prevalence rates, all mixed studies showed higher prevalence in urban compared to rural areas. Estimates from all mixed studies showed an overall prevalence ranging from 17.5% to 51.6% in urban areas and 4.6% to 43% in rural areas. In 5 out of the 6 studies conducted in mixed settings, prevalence were relatively higher in urban than in rural areas. However, only one of the studies [42] reported that prevalence rate was higher in rural compared to urban area.

**With regards to BP cut-off point:** Generally the prevalence differed by wide margins when the BP cut-off point changed from 140/90mmHg to 160/90mmHg. Table 2 shows how prevalence changes with different BP cut-off. Four studies compared different BP cut-off points in their analysis [37,54,56,73]. In all the studies there appears to be a decrease in value of prevalence when BP cut-off was increased.

**Table 2:** Showing changing prevalence (%) using different BP cut-off.

				prevalence rate%(95%CI)	
No	First Author	Year of study	Setting	definition 1	definition 2
**1**	Murthy^73^	2013	national	44.9(43.5–46.3)	24.3(23.6–25.0)
**2**	Cooper^37^	1997	national	14.5(13.1–15.9)	6.9(6.2–7.6)
**3**	Adedoyin^54^	2008	SW	36.6(34.5–38.7)	13.3(11.8–14.8)
**4**	Adebayo,^56^	2013	SW	26.4(23.7–29.1)	11.8(9.8–13.8)

Definition 1: Systolic BP ≥140 mmHg or Diastolic BP ≥ 90 mmHg; Definition 2: Systolic BP ≥ 160 mmHg ≥ or Diastolic BP ≥ 90mmHg

SW = south west

**With regards to trends:** Although pooled estimates were not done in this review, it appears that the mean blood pressure levels may have risen over time. The prevalence figures from the 2 national surveys done in 1997 and 2013 were 11.2% (95% CI: 10.3 to12.1) and 24.3% (95% CI: 23.6 to 25) respectively using BP cut off of ≥160/90mmHg. When a threshold of 140/90mmHg was used in the 2013 national prevalence study, the prevalence rate increased to 45.9% (95% CI: 43.5–46.3%).

### Prevalence of hypertension amongst hospital clients

Only three studies [36,53,69] included in the review were carried out in a hospital setting. Two (2) of the studies [36,53] were conducted in 2009 using a BP cut-off of 140/90mmHg while one (1) [69] was conducted in 1977 with a BP cut off of 160/95mmHg. Crude prevalence recorded in these studies were 3.8% (95% CI: 3.1 to 4.5), 18.4% (95% CI: 17.5 to 19.2) and 30.6% (95% CI: 26.1 to 35.1) conducted in 1977 and 2009 respectively. Although a BP cut-off of 160/95mmHg was used for the 1977 study, the results show a very low prevalence of hypertension among hospital attendees in 1977 compared to later years. This increasing pattern of prevalence rates amongst hospital attendees is similar to that experienced in community based studies.

The hospital based study that revealed a prevalence of 18.4% [36] also showed that during the period of review, 26.5% of all hospital cases and 46.1% of hypertension related complications respectively were due to hypertensive heart failure. A study which reviewed complications of hypertension also showed that the commonest risk factor for stroke, heart failure, and ischemic heart disease and chronic kidney disease in Nigeria was high blood pressure [77].

### Prevalence of hypertension by ethnic groups and Geopolitical zones

There are over 250 distinct ethnic groups in Nigeria. But, only 2 studies have reported prevalence studies by ethnic groups [60,73]. The study by Andy et al [60] compared prevalence of hypertension among only 3 ethnic groups (Obolo, Efiks, Ibibios) resident in the south- south region of the country. The prevalence rates ranged from 14.9% to 25.6% [60]. The lowest rate was recorded amongst the “Obolo” ethnic group while the highest rate was in the “Efiks”.

**Fig 5:**
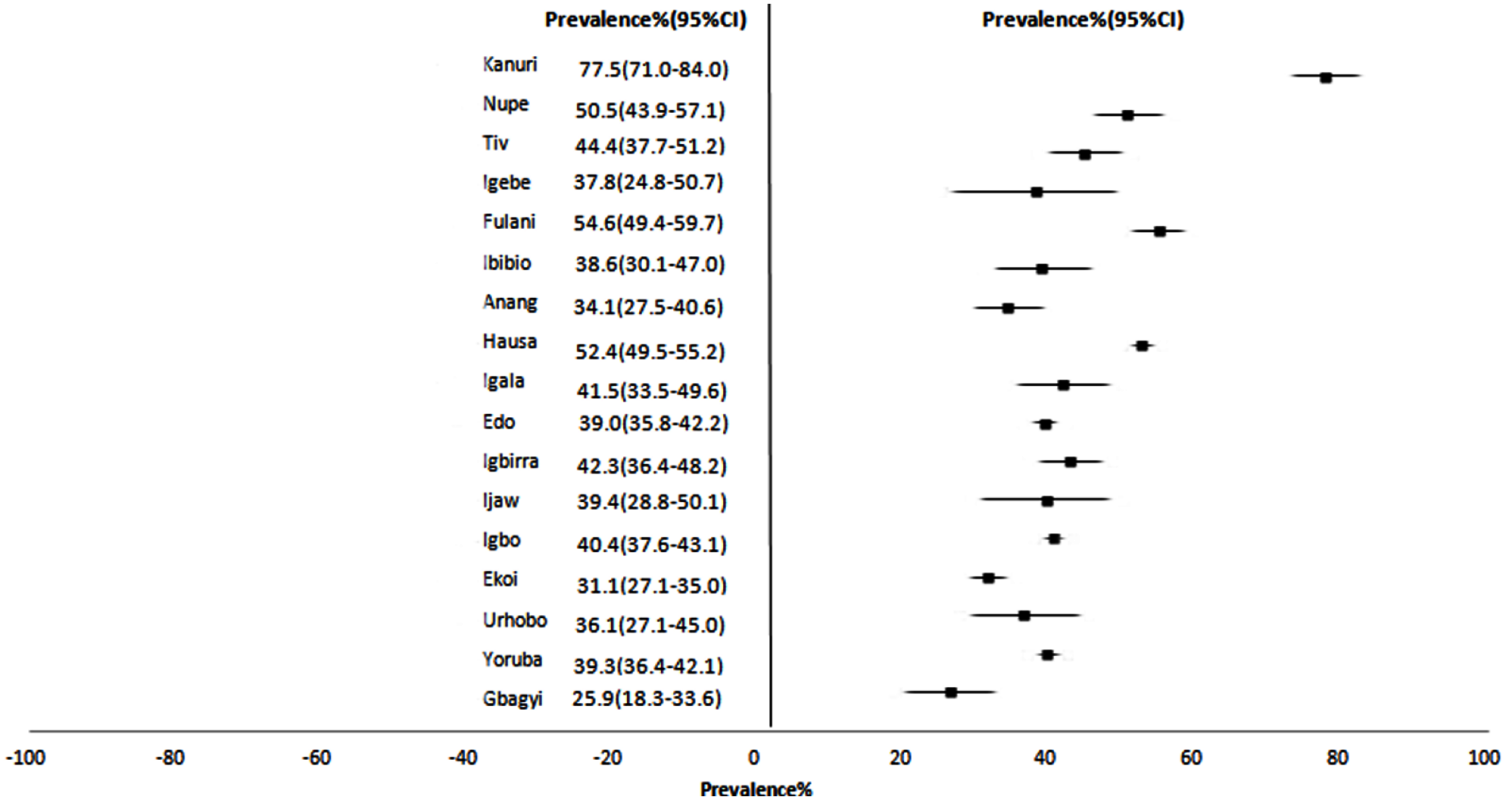
Forest plot showing hypertension prevalence and 95% CI across 17 ethnic groups in the Murthy, G et al 2013 study[73].

But the national survey by Murthy, G. et al [73] cut across 17 major ethnic groups scattered all over the country as illustrated in Fig 5. Using BP cut off of 140/90mmHg, prevalence rates ranged from 25.9% (95%CI: 18.3 to 33.6) to 77.5% (95%CI: 71.0 to 84.0) with the highest being in the Kanuri group and lowest in the Gbagyi group. Prevalence figures obtained among Ibibios(25.5%) in the Andy, J et al [60] study was lesser than in the Murthy, G et al study (38.6%(95%CI:30.1 to 47.0)).

The distribution of hypertension differed highly significantly across ethnic groups in Nigeria, χ^2^ (df, 16) = 361.464, *p* < 0.0001.

In addition, the only study that reported prevalence by geopolitical zone was the national survey by Murthy, G et al [73]. The crude prevalence of hypertension by geopolitical zones (North-east, south-east, south-south, North-west, South-west, North-central) using BP cut off of 140/90mmHg were 60.4% (95%CI:55.1 to 65.8), 41.0%(95%CI:38.0 to 44.0), 34.2%(95%CI:31.2 to 37.2), 51.5%(95%CI:48.7 to 54.2), 40.1%(95%CI:37.1 to 43.2), and 39.5%(95%CI:35.7 to 43.3) respectively. Also, the distribution of hypertension differed highly significantly across geopolitical zones in Nigeria, χ^2^ (df, 5) = 375.656, *p* < 0.0001.

## Discussion

Most studies reported only the crude prevalence of hypertension. Most of the studies carried out in the last 20 years reported a higher prevalence of hypertension compared to older studies [74,79,80]. Reported values of prevalence of hypertension in Nigeria vary widely. These wide variations in values are in part dependent on the blood pressure criteria used. Some of the variations can also be explained by methodological differences (e.g. some have focused on only rural populations) but variation in the age groups studied is likely to be a major factor. Information on trends in the prevalence of hypertension is limited because there were no follow up studies on similar population groups. Although, these populations might be located in the same geo-political zone or state, characteristics of each village or group or tribe differ significantly. A systematic review of hypertension prevalence studies by Adeloye et al[4] observed that pooled prevalence of hypertension increased from 8.6% over the period 1970–1979 to 22.5% over the period 2000–2011.

Many of the studies mentioned in this review that used cut-off of 140/90mmhg reported that prevalence of hypertension was higher in males than females but the reverse was reported for studies that used 160/90mmhg. This pattern is difficult to explain and adequate comparison can only be made with age and sex-adjusted figures which was lacking in most of the included studies. However, the pattern of higher blood pressure in males compared to females of middle age group is similar to findings in other Africans, African Americans and blacks in Caribbean region [81]. Similarly, in a related study, it was shown that men have higher blood pressure than women of the same age group before women attain the age of menopause [82]. This could be due to societal socio-economic roles assigned to men in the home where they have to provide most of the finances for family maintenance [79].

Most of the cross-sectional studies used for this review include middle-age subjects. However, some cross-sectional studies were conducted among older population groups [59,63] or children [27,34,65–67]. Values of Prevalence of hypertension in children are much lower than that reported in adults. Also, there appears to be no gender preponderance for prevalence of hypertension when other risk factors such as obesity have been excluded [27]. Nevertheless, research has shown that blood pressure readings increases with age development and growth and results in high blood pressure by middle-age [83]. In addition, Children with high blood pressure tend to continue to have hypertension as they grow older[84].

The differences in prevalence of hypertension in rural versus urban is evident in most of the included studies. Pooled results from the review done by Adeloye et al also showed a higher urban prevalence compared to rural (31% versus 26%)[4]. This is contextually similar to other studies done in African countries where higher prevalence rates have been reported among urban dwellers [22]. The higher prevalence amongst urban populations may portray a different lifestyle pattern. Urban dwellers are more likely to consume foods that are processed and foods that have high salt and fat content [85]. Furthermore, the lower rural prevalence may indicate higher physical activities levels from trekking long distances and physically taxing farming activities, in addition to greater consumption of freely available vegetables and fruits taking place in majority of rural areas [86, 87]. However, in few studies [42] especially in the eastern part of Nigeria, it was noted that prevalence rate of hypertension was higher in rural than urban area (see Fig 4). This finding is similar to findings in some studies [88–90] in the United States and European population. It is likely that the rural population age pattern is older because it is a popular practice for older people to migrate to rural areas after retirement from active work.

The distribution of hypertension differed among ethnic groups and across geopolitical zones. However, the high hypertension prevalence rate recorded among the Kanuri ethnic group [73] indicates a need for further investigation for possible explanations because this ethnic group is concentrated in only 1 region of the country and the prevalence estimates may be confounded by other factors. Some evidence of ethnic variations has been reported in Kenya with statistically significant differences after adjusting for cardiovascular and socio-demographic risk factors [91].

Report of data from studies done in hospitals reveal that hypertension and cardiovascular disease complications are the commonest NCDs in Nigeria [36,53,69]. This report is very similar to a rate of 30% obtained from Tanzania[92].

A key limitation of this review was that across all studies retained for the review crucial data on sex, age and other descriptive statistics adjusted across rural and urban settings were not always available (see table 1 for overall characteristics of studies included in review).

## Comments

This review has summarised available reports on the prevalence of hypertension in Nigeria. From these reports, it is evident that hypertension is a major public health problem in Nigeria. These prevalence estimates were gleaned from studies conducted in community and hospital based settings. Most of the community based studies were either conducted using house to house surveys or places of mass gathering such as churches, mosques and markets. All of the hospitals were either secondary or tertiary health care centres. However, hypertension studies have not been undertaken specifically on clients who visit primary health care centres which are often the first port of call for people living in both rural and urban areas. This is partly because the focus of primary health care in Nigeria has been on preventive and curative services for endemic communicable diseases and for maternal and child health-related issues in the community.

The effect of non-communicable diseases on development is two-fold [–119–11]. They worsen poverty levels as well as reduce national income available for meaningful development [4,6]. “It is projected that in the next 10 years, China, India and Britain will lose USD 558 billion, USD237 billion, and USD33 billion respectively due to heart disease, stroke and diabetes mellitus”(pg260)[12].

Similarly, in Nigeria the economic burden posed by hypertension and its complications are very high [13]. For example, a study on monthly cost of hypertension treatment per person in a community in the south-western part of Nigeria showed that an average of ten united state dollars (10USD) was spent on drugs alone aside from other direct cost. This is untenable in a population where many live below 2USD a day [4,14].

Moreover, it has been shown that the average monthly cost of treatment of hypertension could be higher especially in cases where the patients have to go back for follow-up more frequently than expected due to complications of management [15].

The low levels of awareness, treatment and control of hypertension, suggest that rates of cardiovascular complications such as cerebro-vascular accidents, heart failure, and renal failure will increase in coming years [4]. As health care services are currently organised in Nigeria, most of these complications will present at secondary and tertiary health care centres and may overwhelm these centres and their resources if measures are not taken to ensure adequate prevention and treatment.

This suggests that there is a need for additional resources for the detection and control of hypertension and other NCDs in addition to resources being allocated for the control of major communicable diseases and neglected tropical diseases. Interventions and strategies will be required to increase adherence to life-style changes and life-long medications. It is clear that hypertension and its complications will cause both governments and societies large financial and societal cost but the government needs to respond to this emerging challenge to ensure the future health of Nigerians.

The implication is that more hypertension and other NCDs strategies should be developed and evaluated specifically in primary health care centres to achieve sustainable policies and practices required for expanding preventive and curative services available at the primary health care level for hypertension and other NCDs in Nigeria.

## Supporting Information

S1 Appendix Search strategy for EMBASE(ovidSP) and MEDLINE(ovidSP). (DOCX)

S2 Appendix Potential studies excluded. (DOC)

S1 PRISMA Checklist PRISMA checklist. (DOC)
