# Supplementary material for: Current Prevalence Pattern of Hypertension in Nigeria: A Systematic Review
Source: PLoS One. 2015 Oct 13;10(10):e0140021. doi: 10.1371/journal.pone.0140021 (PMC4603956; doi:10.1371/journal.pone.0140021)
Supplement: S1 Appendix — (DOCX) [file pone.0140021.s001.docx]

**S1 Appendix**

Search Strategy for **EMBASE (ovidSP) and MEDLINE (ovidSP)**

1. exp epidemiologic methods/ or exp data collection/ or exp health surveys/ or exp health care surveys/ or exp vital statistics/ or exp life expectancy/ or exp life tables/ or exp morbidity/ or exp incidence/ or exp prevalence/ or exp mortality/
2. exp clinical medicine/ or exp community medicine/ or exp global health/ or exp hospital medicine/ or exp internal medicine/ or exp paediatrics/ or exp public health/ or exp epidemiology/ or exp preventive medicine/
3. exp Hypertension/
4. exp Nigeria/
5. (Prevalence$ or incidence$).mp. [mp=title, abstract, original title, name of substance word, subject heading word, keyword heading word, protocol supplementary concept word, rare disease supplementary concept word, unique identifier]
6. (Epidemiology or "vital statistics").mp. [mp=title, abstract, original title, name of substance word, subject heading word, keyword heading word, protocol supplementary concept word, rare disease supplementary concept word, unique identifier]
7. (Blood adj3 pressure).mp. [mp=title, abstract, original title, name of substance word, subject heading word, keyword heading word, protocol supplementary concept word, rare disease supplementary concept word, unique identifier]
8. hypertension.mp. [mp=title, abstract, original title, name of substance word, subject heading word, keyword heading word, protocol supplementary concept word, rare disease supplementary concept word, unique identifier]
9. ("high blood pressure" or "increased blood pressure").mp. [mp=title, abstract, original title, name of substance word, subject heading word, keyword heading word, protocol supplementary concept word, rare disease supplementary concept word, unique identifier]
10. Nigeria$.mp. [mp=title, abstract, original title, name of substance word, subject heading word, keyword heading word, protocol supplementary concept word, rare disease supplementary concept word, unique identifier]
11. 1 or 2
12. 5 or 6
13. (7 or 8) not 9
14. 11 or 12
15. 3 or 13
16. 4 or 10
17. 14 and 15 and 16
18. limit 17 to humans
