# Supplementary material for: Current Prevalence Pattern of Hypertension in Nigeria: A Systematic Review
Source: PLoS One. 2015 Oct 13;10(10):e0140021. doi: 10.1371/journal.pone.0140021 (PMC4603956; doi:10.1371/journal.pone.0140021)
Supplement: S2 Appendix — (DOC) [file pone.0140021.s002.doc]

**S2 Appendix**

| **POTENTIAL STUDIES EXCLUDED** | | | |
| --- | --- | --- | --- |
|  |  |  |  |
| **NO** | First Author | Year of study | Reason for Exclusion |
| **1** | Ahaneku et al | 2011 | sample size < 400 |
| **2** | Adeloye, D et al | 2015 | editorial and review |
| **3** | Tovagirumukiza,M et al | 2011 | editorial and review |
| **4** | Opie, L et al | 2005 | editorial and review |
| **5** | Hult, M et al | 2000 | point prevalence not calculated |
| **6** | Bello, M et al | 2013 | editorial and review |
| **7** | Ekwunife, I et al | 2011 | editorial and review |
| **8** | Ordinioha, B et al | 2011 | sample size < 400 |
| **9** | Adamu, U. et al | 2013 | point prevalence not calculated |
| **10** | Oduwole,A et al | 2012 | point prevalence not calculated |
| **11** | Ogunmola, O. et al | 2013 | sample size < 400 |
| **12** | Mezue, K et al | 2014 | editorial and review |
| **13** | Adediran, O.et al | 2013 | point prevalence not calculated |
| **14** | Forester,T et al | 1998 | editorial and review |
| **15** | Din-Dzietham, R et al | 2007 | editorial and review |
| **16** | Ejike, C et al | 2008 | point prevalence not calculated |
| **17** | Balogun,I et al | 1990 | point prevalence not calculated |
| **18** | Hamidu,L et al | 2000 | point prevalence not calculated |
| **19** | Akinkugbe, F et al | 1999 | point prevalence not calculated |
| **20** | Kaufman, J et al | 1996 | point prevalence not calculated |
| **21** | Cooper, R et al | 1998 | editorial and review |
| **22** | Bosu,W et al | 2010 | editorial and review for Ghana |
| **23** | Oghagbon, C et al | 2008 | sample size < 400 |
| **24** | Kearney,P et al | 2004 | editorial and review |
| **25** | Oghenekaro,G et al | 2012 | sample size < 400 |
| **26** | Taylor,G et al | 1996 | point prevalence not calculated |
| **27** | Boutayeb,A et al | 2006 | editorial and review |
| **28** | Unwin,N et al | 2001 | editorial and review |
| **29** | Ekere, A et al | 2005 | point prevalence not calculated |
| **30** | Ike, S et al | 2008 | sample size < 400 |
| **31** | Sani, M et al | 2010 | sample size < 400 |
| **32** | Ofuya, I | 2007 | sample size < 400 |
| **33** | Addo, J et al | 2007 | editorial and review |
